# HIF-1 signalling pathway was identified as a potential new pathway for Icariin’s treatment against Alzheimer’s disease based on preclinical evidence and bioinformatics

**DOI:** 10.3389/fphar.2022.1066819

**Published:** 2022-12-01

**Authors:** Mingyao You, Ping Yuan, Liangqian Li, Hongbei Xu

**Affiliations:** Department of Neurology, The Affiliated Hospital of Guizhou Medical University, Guiyang, China

**Keywords:** Icariin, Alzheimer’s disease, literature review, network pharmacology, HIF-1 signalling pathway

## Abstract

**Aim:** Alzheimer’s disease (AD) is a neurodegenerative condition that is characterized by the gradual loss of memory and cognitive function. Icariin, which is a natural chemical isolated from *Epimedii herba*, has been shown to protect against AD. This research examined the potential mechanisms of Icariin’s treatment against AD *via* a comprehensive review of relevant preclinical studies coupled with network pharmacology.

**Methods:** The PubMed, Web of Science, CNKI, WANFANG, and VIP databases were used to identify the relevant studies. The pharmacological characteristics of Icariin were determined using the SwissADME and TCMSP databases. The overlapping targets of Icariin and AD were then utilized to conduct disease oncology (DO) analysis to identify possible hub targets of Icariin in the treatment of AD. The hub targets were then used for Gene Ontology (GO) and Kyoto Encyclopedia of Genes and Genomes (KEGG) pathway enrichment analyses, and the interactions of the targets and Icariin were assessed *via* molecular docking and molecular dynamics simulation (MDS).

**Results:** According to the literature review, Icariin alleviates cognitive impairment by regulating the expression of Aβ_1-42_, Aβ_1-40_, BACE1, tau, hyperphosphorylated tau, and inflammatory mediators. DO analysis revealed 35 AD-related hub targets, and the HIF-1 signalling pathway was ranked first according to the KEGG pathway analysis. Icariin effectively docked with the 35 hub targets and HIF-1α, and the dynamic binding of the HIF-1-Icariin complex within 100 ns indicated that Icariin contributed to the stability of HIF-1α.

**Conclusion:** In conclusion, our research used a literature review and network pharmacology methods to identify the HIF-1 signalling pathway as a potential pathway for Icariin’s treatment against AD.

## 1 Introduction

Alzheimer’s disease (AD) is a progressive neurological disorder that mostly affects elderly individuals and causes memory loss and other cognitive impairments (Corbo et al., 2021). More than 50 million individuals throughout the globe are affected by this illness, which causes significant burden on current public health systems (Opara et al., 2017). Amyloid-β (Aβ) plaques, neurofibrillary tangles (NFTs, composed of phosphorylated tau), and synaptic loss are recognized as the primary hallmarks of AD (Breijyeh and Karaman, 2020; Ashrafian et al., 2021), although the underlying aetiology is still not completely understood. Patients with this condition experience symptoms and dysfunction owing to neuronal loss, which is caused by different variables that have been implicated in the pathogenesis of AD. To date, AD is among the least well-served therapeutic areas for drug treatments. Employing agents that target the pathological mechanisms underlying this disease in order to delay its development is of great importance (Cummings et al., 2019; Breijyeh and Karaman, 2020).

Icariin, which is a type of flavonoid, is the main ingredient that is extracted from *Epimedium* (Jin et al., 2019). Multiple pharmacological activities, such as antioxidant, anti-inflammatory, and antiapoptotic activities, have been attributed to Icariin, which may account for the compound’s purported preventative and therapeutic efficacy in conditions as diverse as ischaemic stroke (Liu et al., 2018; Dai et al., 2021; Wu et al., 2021), AD (Ma et al., 2021; Wang et al., 2022; Yan et al., 2023), Parkinson’s disease (Lu et al., 2018; Zeng et al., 2019; Khezri and Ghasemnejad-Berenji, 2022), multiple sclerosis (Shen et al., 2015; Cong et al., 2020), and depressive disorder (Cao et al., 2019; Xu et al., 2020; Zeng et al., 2022). Several studies have demonstrated that Icariin treatment can suppress Aβ production to improve learning and memory in animals (Jin et al., 2014a; Chen et al., 2016a; Chuang et al., 2021). However, the pharmacological properties of Icariin and the molecular basis for its effects on AD are not fully understood.

Network pharmacology is a systematic and comprehensive research strategy that is used to predict the mechanisms by which drug treatments affect illnesses, and it involves a “network-target, multiple-component-therapeutics” approach (Zhang et al., 2019a). Molecular docking is used to predict the binding mode and affinity of receptors and the mode of interaction between receptors and drug moleculars (Pinzi and Rastelli, 2019). By combining drug target networks with biological system networks, network pharmacology facilitates novel approaches to drug discovery. However, network pharmacology has not been used to explore the neuroprotective role of Icariin in AD. In the current study, a literature review combined with network pharmacology was used to systemically analyse the neuroprotective role of Icariin in AD and to comprehensively predict the possible mechanisms. The flow chart of this study was shown in Supplementary Material S1.

## 2 Material and methods

### 2.1 The meta-analysis

This study conducted a comprehensive literature search to identify appropriate studies that described improved learning and memory functions in rodent animal AD models after Icariin treatment. We collected the relevant literature from five separate databases, namely, PubMed, Web of Science, CNKI database, Wanfang database, and VIP database, and the published languages were either English or Chinese. The period for the literature review was from the establishment of the database to August 2022. The strategies for article retrieval are shown in Supplementary Material S2. The quality of each study was independently evaluated by two researchers.

#### 2.1.1 Inclusion and exclusion criteria

##### 2.1.1.1 Inclusion criteria

1) AD rodent animal models regardless of the species, age, sex, or weight of the animals.
2) The experimental group was treated with Icariin. A control group treated with a placebo, such as saline or similar vehicles, was also needed. The doses, administration methods, and duration of treatment were not limited.
3) The study was an original experimental study of the effects of Icariin on animal models of AD.

##### 2.1.1.2 Exclusion criteria

1) Literature containing incorrect or missing information; duplicate references; review articles; absence of complete text.
2) Icariin-related studies were not conducted in rodent models of AD.
3) Icariin was used in conjunction with other medications or treatments.
4) The study had poor quality according to an assessment of the experimental design, the reliability of the findings, and the reputation of the journals.

#### 2.1.2 Data extraction and analysis

The graphs in each study were extracted *via* OriginPro software to obtain numerical values. A meta-analysis performed with Review Manager 5.4 was used to evaluate some of the results. The risk of bias (ROB) of the included publications was assessed using SYRCLE’s risk of bias instrument for animal studies (Krauth et al., 2013).

### 2.2 Network pharmacology

#### 2.2.1 Identification of the pharmacological and potential targets of icariin

The SwissADME database (http://www.swissadme.ch) was used to search for the pharmacological properties of Icariin. The Traditional Chinese Medicine Systems Pharmacology (TCMSP, http://tcmspw.com/tcmsp.php) database was used to evaluate the blood–brain barrier (BBB) score of Icariin. The 2D and 3D structures of Icariin were acquired from the PubChem database (https://pubchem.ncbi.nlm.nih.gov/). The canonical SMILES of Icariin is CC1C(C(C(C(O1)OC2 = C(OC3 = C(C2 = O)C (=CC(=C3CC = C(C)C)OC4C(C(C(C(O4)CO)O)O)O)O)C5 = CC = C(C=C5)OC)O)O)O, which was computed in the PubChem database.

Drug toxicology is one of the key fields of preclinical research. The toxicological parameters of Icariin were identified *via* the Protzox II webserver (https://tox-new.charite.de/protox_II/). The toxicological end points were described in a binary format as active or inactive.

The potential targets of Icariin were predicted with TCMSP, Comparative Toxicogenomics Databases (CTD, http://ctdbase.org/), Therapeutic Target Database (TTD, http://db.idrblab.net/ttd/), Similarity ensemble approach (SEA, https://sea.bkslab.org/) database, and Swiss Target Prediction (STP, http://www.swisstargetprediction.ch/) database.

#### 2.2.2 Screening of AD-related targets

The potential targets for AD were extracted from the GeneCards database (https://www.genecards.org), Online Mendelian Inheritance in Man (OMIM) database (https://omim.org), and Disgenet database (https://www.disgenet.org/home), with “Alzheimer”s disease as the key word. Targets retrieved from the GeneCards database were further filtered with a relevance score ≥10.

#### 2.2.3 Protein‒protein interaction network construction and analysis

The overlapping targets between Icariin and AD were identified *via* the Venny 2.1 platform (https://bioinfogp.cnb.csic.es/tools/venny). The R packages “Disease Ontology Semantic and Enrichment analysis (DOSE)” and “org.Hs.eg. Db” were used to analyse the disease oncology of the intersection targets. In the current study, a PPI network was constructed *via* the STRING11.0 database (https://string-db.org/cgi/), and the biological species was set to “*Homo sapiens*.” The minimum required interaction score was set to “highest confidence>0.900,” and all the remaining settings were set to default. Cytoscape 3.9.1 software was further used to analyse the topological characteristics of the targets.

#### 2.2.4 Analysis of AD-related targets in the alzdata database

The AlzData (http://www.alzdata.org/) database contains an entire collection of current high-throughput omics data for AD. The function of Single Cell Expression in AlzData was used to analyse the cellular distribution of the targets in the human brain based on data derived from the GEO database (GSE67835). The Aging Atlas (https://ngdc.cncb.ac.cn/aging/index) is a database that is used to examine the genetic relationship between lifespan and ageing. The AD-related targets were imported into this database to identify the ageing-related targets.

#### 2.2.5 Gene Ontology and Kyoto Encyclopedia of Genes and Genomes pathway enrichment analyses

The enrichment of GO and KEGG pathways was analysed using the R packages “ClusterProfiler” and “org.Hs.eg. Db” to identify the underlying biological function (BP), cellular component (CC), molecular function (MF), and signalling pathways. The threshold for statistical significance was set to p. Adjust (FDR) 0.05. For the GO and KEGG enrichment findings, Sangerbox (http://sangerbox.com/Tool) was used to create the bars and circular circles.

#### 2.2.6 Molecular docking and molecular dynamics simulation

Crystal structures of hub targets were acquired from the RCSB Protein Data Bank (http://www.rcsb.org/), and three-dimensional structures of Icariin were downloaded from the PubChem database. Icariin served as the ligand, while the central targets served as the receptors. The PDB ID of each protein was provided in Supplementary Material S5. Water molecules and heteroatoms were removed from the receptors using PyMOL 2.4 (https://pymol.org/2/) and AutoDock 4.2.6 (http://autodock.scripps.edu/), and then, charges and hydrogen atoms were added using these programs. Binding conformations between ligands and receptors were then predicted using AutoDockTools. The likelihood of ligand‒receptor binding was calculated using the binding energy (usually −5 kcal/mol). The lower the binding energy was, the greater the molecular docking effect was.

We then used MDS to confirm the stability of the Icariin-HIF-1α complex and obtain a deeper understanding of the dynamic behaviour of Icariin in the HIF-1α active region. The partial atomic charges were generated using the general AMBER force field, and the substrates were built using the restricted electrostatic potential (RESP) method (Wang et al., 2004). MDS was carried out using the AMBER 14sb force field after the complex was solvated in a TIP3P rectangular box that extended 10 from the protein’s border. Complete minimization of the original complex was achieved by iteratively annealing the solution from 10 to 300 K in a classical ensemble for a total of 0.5 ns Using a Langevin thermosta and Berendsen barostat, we were able to achieve density equilibration in 500 ps (Berendsen et al., 1984; Larini et al., 2007) by setting the collision frequency to 0.002 ns and the pressure-relaxation period to 0.001 ns After performing the necessary minimizations and equilibrations, an efficient 100 ns molecular dynamics run for the Icariin-HIF-1α complex was obtained. Within the gmx mmpbsa community, GROMACS MM/PB(GB)SA was used to compute MDS (Valdes-Tresanco et al., 2021).

## 3 Results

### 3.1 Study selection

**FIGURE 1:**
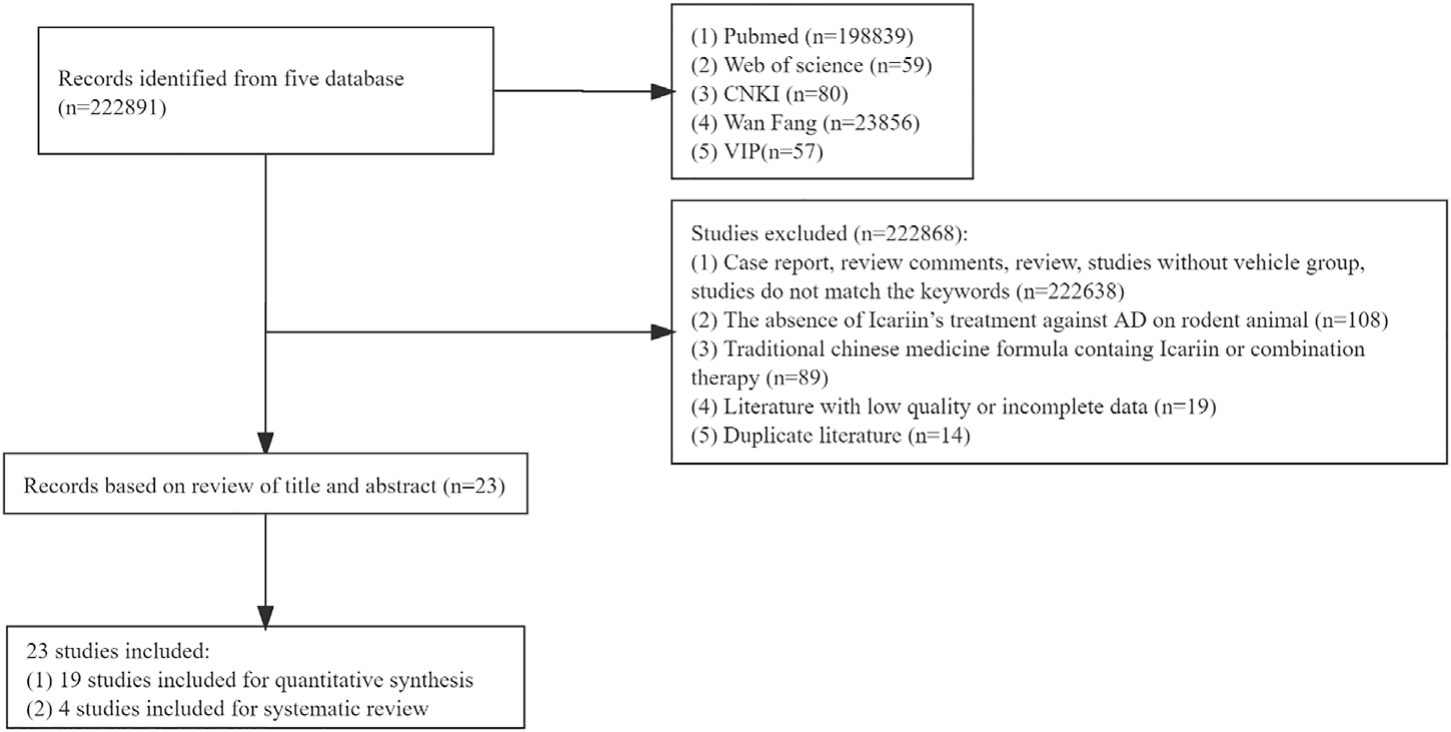
Flow chart of the meta-analysis.

A total of 222891 studies were collected from each database. Following an assessment of the full contents, 222868 papers were eliminated for at least one of the following reasons: 1) the study was case report, review comments, review, study without vehicle group, or study that did not match the key words (*n* = 222638); 2) the study did not include the use of Icariin to treat AD in a rodent model (*n* = 108); 3) the treatment was a traditional Chinese medicine formula containing Icariin or combination therapy (*n* = 89); 4) study had low-quality or incomplete data (*n* = 19); and 5) the study was a duplicate (*n* = 14). Finally, 23 studies were included, 19 studies were used for meta-analysis, and 4 studies were used for systematic review as well. The flow chart of the meta-analysis was illustrated in Figure 1.

### 3.2 The main information extracted from the included studies

The primary data extracted from the 23 included studies are shown in Table 1. Two animal species were included: rat (*n* = 7) and mouse (*n* = 16). There were 16 articles in which male animals were used, 4 articles in which both female and male animals were used, 2 articles in which female animals were used and 1 article did not mention which the sex of animals that were used. Twelve strains were used: Wistar rats (*n* = 1), senescence-accelerated mouse strain prone 8 (SAMP8) mice (*n* = 3), hybridization between APP/PS1-21 mice and C57BL/6J mice (*n* = 1), APP/PS1 mice (*n* = 4), 3 × Tg-AD mice (*n* = 2), hybridization between 3 × Tg-AD mice and WT mice (n = 1), hybridization between Tg2576 mice and C57B6/SJL mice (n = 1), APPV717I mice (n = 1), SAMP10 mice (n = 1), SD rats (n = 6), hybridization between 5xFAD mice and B6/SJLF1 mice (n = 1), and C57BL/6 mice (n = 1).

**TABLE 1:** The detailed information of each study.

First author, year	Animal data	Icariin administration (i.g.: By gavage; p.o: By oral route)	Items evaluated to explore the targeting effects and mechanisms
Wu et al. (2020)	SAMP8 mice and SAMR1 mice (male, 6 months, 19–23 g)	Dosage: 60 mg/kg; Ad: i.g.; Duration: 22 days	Improvement in cognitive impairment; Protein expression level (BACE1↓, Aβ_1-42_↓, Bcl-2/Bax ratio↑, Bax↓)
Li et al. (2015)	AD model was prepared by hybridization between T g2576 male mice and C57B6/SJL female mice (9 months)	Dosage: 60 mg/kg/d; Ad: p.o.; Duration: 3 months	Protein expression level (Aβ_1-40_↓, Aβ_1-42_↓, APP↓, proportion of BrdU^+^NeuN^+^ cells↑)
Jin et al. (2014a)	APP/PS1 mice and WT mice (male, 10 months)	Dosage: 60 mg/kg, Bid; Ad: i.g.; Duration: 4 months	Improvement in cognitive impairment; Protein expression level (Aβ_1-40_↓, Aβ_1-42_↓, APP↓, PDE5↓, nNOS↑, iNOS↑, eNOS↑); NO-cGMP signalling pathway (NO↑, cGMP↑)
Chen et al. (2016a)	3×T g-AD mice (female, 2 months)	Dosage: 65 mg/kg/d; Ad: i.g.; Duration: 6 months	Improvement in cognitive impairment; Protein expression level (BACE1↓, Aβ_1-42_↓, PDHE1a↑, COX↑, PSD95↑; ATP↑); Levels of neurometabolites (NAA/Cr↑; MI/Cr↑; Lac/Cr↓)
Zhang et al. (2014a)	APPV717 mice (male and female, 3 months)	Dosage: 0.01 ml/g weight; Ad: i.g.; Duration: 6 months	Improvement in cognitive impairment; Protein expression level (APP↓, BACE1↓, Aβ_1-42_↓, amyloid plaques↓)
Nie et al. (2010)	AD model was established by injection of Aβ25-35 in Wistar mice (male, 6 months, 400–600 g)	Dosage: 30, 60, 120 mg/kg; Ad: i.g.; Duration: 14 days	Improvement in cognitive impairment; mRNA Expression level (Aβ_1-40_↓, β-secretase↓, SOD-2↑)
Zhu et al. (2019)	C57BL/6 mice and APP/PS1 mice (male, 4 months)	Dosage: 60 mg/kg/d; Ad: i.g.; Duration: 8 months	Improvement in cognitive impairment; Protein expression level (Aβ_1-40_↓, Aβ_1-42_↓, IL-1β↓); Inflammatory cytokines (IL-1β↓, TNF-α↓, IFN-γ↓, MCP-1↓, IL-17A↓, GM-CSF↓, IL-12p70↓, MCP-1↓, IL-10↑)
Gao et al. (2012)	SAMP10 mice and SAMR1 mice (male and female, 8 months)	Dosage: 50, 100, 200 mg/kg; Ad: i.g.; Duration: 30 days	Improvement in cognitive impairment; Protein expression level (Ach↑, MCBC↑, ChAT↑)
Jiang et al. (2019)	AD model was established by injection of Aβ_1-42_ in SD mice (male, 3–4 months, 200–250 g)	Dosage: 30, 60, 90 mg/kg/d; Ad: p.o.; Duration: 4 weeks	Improvement in cognitive impairment; Protein expression level (APP↓, Aβ_1-42_↓, LC3-II/LC3-I↓, Beclin1↓, Cat D↓, SOD↑, Cleaved-caspase-3↓, pS473AKT/AKT ratio↑, GFAP↓)
Yan et al. (2023)	AD model was prepared by hybridization between 3×Tg-AD mice and WT mice (male, 3 months)	Dosage: 60 mg/kg; Ad: i.g.; Duration: 5 months	Improvement in cognitive impairment; Protein expression level (NeuN↑, PSD95↑, Aβ_1-40_↓, Aβ_1-42_↓, APP↓, hyperphosphorylated tau at the Thr217, Ser199/202 and Thr231, Ser396/404 sites↓, GLUT1↑, GLUT3↑); Restored impaired insulin signalling
Zhang et al. (2012)	SAMP8 mice and SAM-R mice (male, 6 months, 21.5–27.5 g)	Dosage: 0.01 ml/g; Ad: i.g.; Duration: 8 months	Improvement in cognitive impairment; Cell proportions (p-CREB-positive cells↑, CREB phosphorylation↑)
Zhang et al. (2014b)	Hybrid male APP/PS1-21 mice were mated with wild type C57BL/6J female mice to prepare AD model (6 males and 8 females, 5 months)	Dosage: 100 mg/kg; Ad: i.g.; Duration: 10 days	Protein expression level (Aβ plaque counts and area↓, TGF-β1↓, lba-1^+^ area↓, GFAP-IR area↓, TGF-b1 IR plaque↓)
Sheng et al. (2017)	AD model was established by injection of Aβ_1-42_ in SD mice (male, adult, 200–250 g)	Dosage: 30, 60, 120 mg/kg; Ad: i.g.; Duration: 28 days	Improvement in cognitive impairment; Improvement in synaptic ultrastructure; Cell proportions (BDNF↑, pCREB↑); Protein expression level (PSD-95↑, pTrkB/TrkB ratio↑); mRNA expression (BDNF↓, TrkB↓, CREB↓)
Urano and Tohda, (2010)	AD model was prepared by hybridization between 5xFAD mice and B6/SJLF1 mice (male, 9 months)	Dosage: 50 μmol/kg; Ad: p.o.; Duration: 8 days	Improvement in cognitive impairment; Increased axon and dendrite length
Wang et al. (2020a)	C57BL/6 mice and APP/PS1 mice (male, 4 months)	Dosage: 60 mg/kg/d; Ad: i.g.; Duration: 8 months	Improvement in cognitive impairment; Protein expression level (Aβ↓); Positive staining area (GFAP↓, Iba-1↓, IL-1β↓); Inhibition of NF-κB p65 phosphorylation in brain
He et al. (2022)	AD model was established by injection of Aβ_1-42_ in SD mice (male, 200–240 g)	Dosage: 0.03, 0.06, 0.09 g/kg/d; Ad: i.g.; Duration: 4 weeks	Improvement in cognitive impairment; Protein expression level (IL-1β↓, IL-6↓, TNF-α↓, RhoA↓, ROCK1↓, ROCK2↓); Improvement in the pathological morphology of hippocampus; Increased dendritic spine density and dendritic length; mRNA expression levels (TNF-α↓, IL-1β↓, IL-6↓, RhoA↓, ROCK1↓, ROCK2↓)
Li et al. (2014)	AD model was established by injection of ICV- STZ in SD mice (male, 280–320 g)	Dosage: 40 mg/kg/d; Ad: i.g.; Duration: 6 weeks	Improvement in cognitive impairment; Protein expression level (AchE↓, TGF- β1↓); ICV-STZ did not affect the blood glucose level
Li et al. (2019a)	AD model was established by injection of Aβ in C57BL/6 mice (male, 28–32 g, adult)	Dosage: 30 mg/kg; Ad: i.g.; Duration: 20 days	Improvement in cognitive impairment; Protein expression level (Phosphorylated tau↓, mPR↓, c-Jun↓)
Chen et al. (2017)	AD model was established by injection of Aβ_1-42_ in SD mice (280–300 g0)	Dosage: 60, 120 mg/kg; Ad: i.g.; Duration: 30 days	Improvement in cognitive impairment; Protein expression level (ZO-1↑, MMP-9↓)
Li et al. (2019b)	APP/PS1 trans genic mice and WT mice (male, 35–45 g, 1 month)	Dosage: 60 mg/kg; Ad: p.o.; Duration: 3 months	Improvement in cognitive impairment; Protein expression level (Aβ_1-40_↓, Aβ_1-42_↓, APP↓, BACE1↓, ADAM10↑); Inhibit ER stress in APP/PS1 mice by suppressing the PERK/eIF2α signalling pathway; Reduced apoptosis by ICA treatment mice was partly due to suppressed ER stress
Yu et al. (2022)	3×T g-AD mice harbouring the APPSwe, TauP301L and PSEN1M146V Trans genes, WT B6 mice (female, 4 months)	Dosage: 120 mg/kg; Ad: p.o.; Duration: 4 months	Improvement in cognitive impairment; Protein expression level (Aβ_1-42_↓, tau↓); Alleviated Myelin Sheath Damage; Increased Expression of Myelin-Related Gene
Jin et al. (2014b)	AD model was established by injection of Aβ_25-35_ in SD mice (male, 230–270 g, 2–3 months)	Dosage: 10 mg/kg; Ad: i.g.; Duration: 4 weeks	Protein expression level (SYN↑, PSD-95↑); Improvement in the histological morphology of the hippocampus
Chen et al. (2019)	SAMP8 mice and SAMR1 mice (male, 5 months)	Dosage: 20, 40, 80 mg/kg; Ad: i.g.; Duration: 3 months	Improvement in cognitive impairment; Senescence-associated β-galactosidase activity↓; Inhibition of hippocampal neuron loss and reversal of hippocampal neuron structural changes; Decreased number of autophagosomes, accompanied by organized endoplasmic reticulum in hippocampal neurons; Protein expression level (LC3-II↓, p62↓)

### 3.3 Analysis of the effects of icariin treatment in AD

#### 3.3.1 The effect of icariin treatment on cognitive function, Aβ1-42 deposition, APP and BACE1 expression in AD animals

AD patients exhibited enhanced extracellular β-amyloid protein levels, Aβ deposition, abnormal phosphorylation of tau protein, neuronal loss, neurodystrophy, and synaptic formation. Changes in learning and memory functions, Aβ deposition, APP protein levels, BACE1, p-tau and related inflammatory mediators are critical indicators of the pathology and severity of AD and contribute to evaluating the therapeutic effect.

**FIGURE 2:**
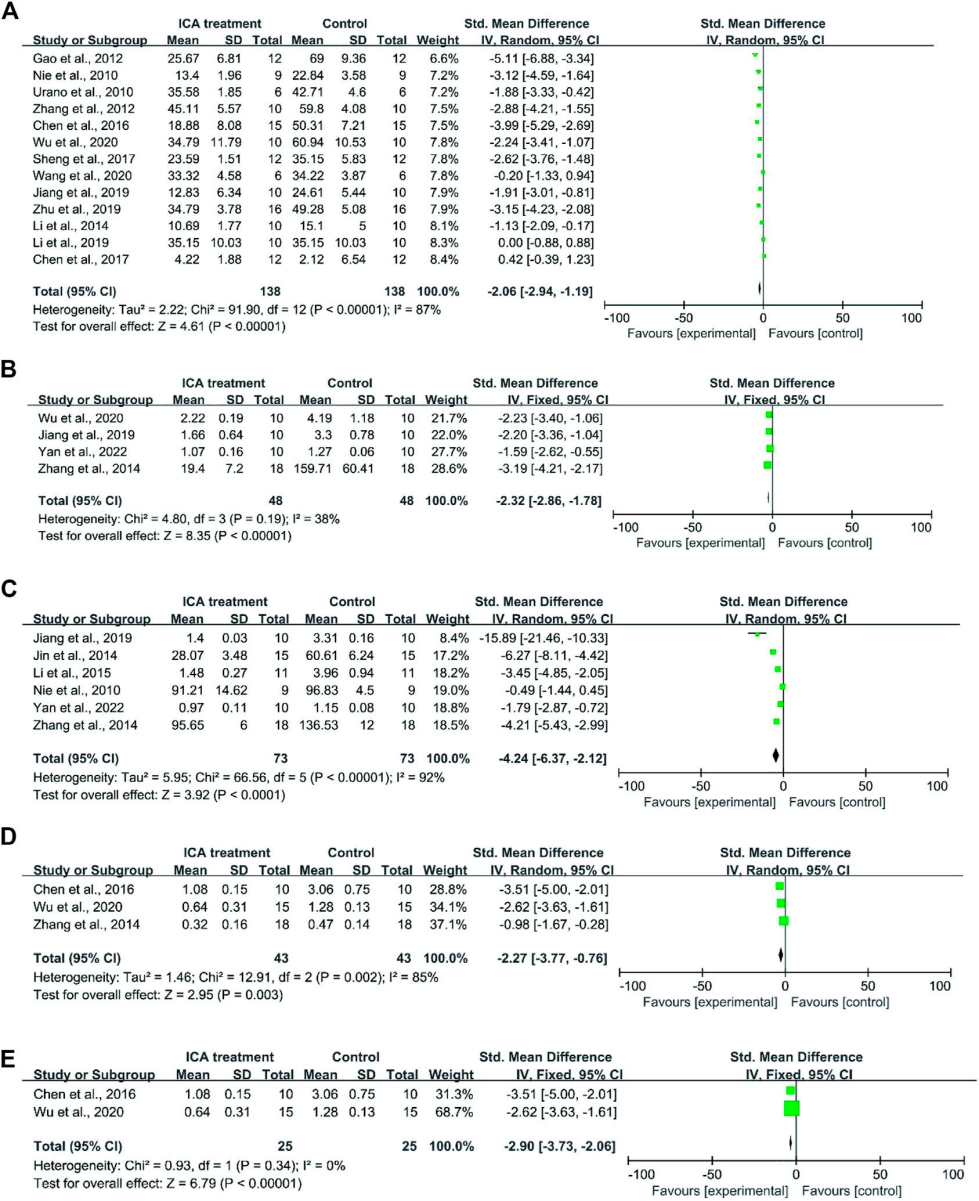
Forest plot for each indicator. **(A)** Forest plot indicated that Icariin treatment significantly improved the cognitive function of AD animals. **(B)** Forest plot of Aβ_1-42_ deposition indicated that Icariin significantly suppressed Aβ_1-42_ protein deposition. **(C)** Forest plot indicated that Icariin inhibited APP expression. **(D)** Icariin treatment inhibited BACE1 expression before sensitivity analysis (I^2^ = 85%). **(E)** Icariin treatment inhibited BACE1 expression after sensitivity analysis (I^2^ = 0%).

First, cognitive function was evaluated based on the data that were extracted from 8 studies. A total of 73 mice were treated with Icariin and vehicle [Nie et al. (2010); Urano and Tohda (2010); Zhang et al. (2012); Wu et al. (2020); Sheng et al. (2017); Wang et al. (2020a); Jiang et al. (2019); Li et al. (2014)] (Figure 2A). Icariin treatment significantly improved the learning and memory function of AD animals (I^2^ = 87%, *p* < 0.00001). After sensitivity analysis was performed by removing the relevant studies one by one due to the high heterogeneity, the results were consistent (Table 2).

**TABLE 2:** The sensitivity analysis for cognitive function

Items	Eliminated literature	1^2^ (%)	SMD	95% CI
**cognitive function**	Gao et al. (2012)	86	−5.11	[−6.88, −3.34]
	Nie et al. (2010)	87	−3.12	[−4.59, −1.64]
	Urano and Tohda, (2010)	88	−1.88	[−3.33, −0.42]
	Zhang et al. (2012)	88	−2.88	[−4.21, −1.55]
	Chen et al. (2016a)	86	−3.99	[−5.29, −2.69]
	Wu et al. (2020)	88	−2.24	[−3.41, −1.07]
	Sheng et al. (2017)	88	−2.62	[−3.76, −1.48]
	Wang et al. (2020a)	87	−0.2	[−1.33, 0.94]
	Jiang et al. (2019)	88	−1.91	[−3.01, −0.81]
	Zhu et al. (2019)	87	−3.15	[−4.23, −2.08]
	Li et al. (2014)	88	−1.13	[−2.09, −0.17]
	Li et al. (2019a)	86	0	[−0.88, 0.88]
	Chen et al. (2017)	83	0.42	[−0.39, 1.23]
**APP expression**	Jiang et al. (2019)	91	−15.89	[−21.46, −10.33]
	Jin et al. (2014a)	92	−6.27	[−8.11, −4.42]
	Li et al. (2015)	94	−3.45	[−4.85, −2.05]
	Nie et al. (2010)	90	−0.49	[−1.44, 0.45]
	Yan et al. (2023)	94	−1.79	[−2.87, −0.72]
	Zhang et al. (2014a)	93	−4.21	[−5.43, −2.99]
**BACE1 expression**	Wu et al. (2020)	86	−3.51	[−5.00, −2.01]
	Chen et al. (2016a)	89	−2.62	−3.63, −1.61]
	Zhang et al. (2014a)	0	−0.98	[−1.67, −0.28]

For the analysis of Aβ_1-42_ deposition, a total of five studies were included for the assessment of Aβ_1-42_ deposition (Wu et al. (2020); Jiang et al. (2019); Yan et al. (2023); Zhang et al. (2014a)). The result indicated that Icariin treatment significantly suppressed Aβ_1-42_ protein deposition (I^2^ = 38%, *p* < 0.0001, Figure 2B).

For APP analysis, six studies were included (Jiang et al. (2019); Li et al. (2015); Jin et al. (2014a); Zhang et al. (2014a); Yan et al. (2023); Nie et al. (2010)), and the results indicated that Icariin treatment could inhibit APP expression. Due to the high heterogeneity (I^2^ = 92%, Figure 2C) resulting from the different methods of analysis, sensitivity analysis was performed by eliminating the studies one by one (Table 2). The sensitivity analysis indicated that there was no significant change in heterogeneity, and the difference was still statistically significant (*p* < 0.0001), which means that these findings were stable.

The levels of BACE1 expression from four studies (Chen et al., 2016a); Wu et al. (2020); Zhang et al. (2014a) were included in the meta-analysis and exhibited high heterogeneity (I2 = 85%, *p* = 0.003) (Figure 2D). In the sensitivity analysis (Table 2), the heterogeneity changed from 85% to 0% after Zhang et al. (2014a) was excluded. The reason for the heterogeneity is that the research of Zhang et al. (2014a) analysed the ratio of BACE1 expression between the experimental group and the control group, while the studies of Chen et al. (2016a) and Wu et al. (2020)) used the ratio of BACE1/loading control to analyse BACE1 expression. Icariin treatment still significantly decreased BACE1 expression after sensitivity analysis (*p* < 0.00001, Figure 2E).

#### 3.3.2 Icariin treatment suppressed the inflammation induced by AD

The formation of NFTs in the brains of AD patients occurs due to the hyperphosphorylation of the tau protein, which can be phosphorylated at multiple sites (Kinney et al., 2018). Yan et al. (2023) showed that Icariin treatment significantly reduced tau hyperphosphorylation at the Thr217, Ser199/202, Thr231, and Ser396/404 sites in AD mice. The pathophysiology of AD involves not only the accumulation of aberrant protein aggregates but also neuroinflammation (Gao et al., 2022). Glial cells are the primary immune cells in the brain and exert beneficial and neuroprotective effects by removing pathogens. However, growing evidence suggests that the homeostatic function of these glial cells is drastically diminished in AD (Watroba and Szukiewicz, 2022). The studies included in our work indicated that Icariin treatment could inhibit the release of the proinflammatory cytokines IL-1 β, IL-6, and TNFα by regulating the NF-kB signalling pathway Wang et al. (2019); Zhang et al. (2014b); Wang et al. (2020a); He et al. (2022)) and promote the expression of the anti-inflammatory cytokines IL-4, IL-10 and transforming growth factor β (TFG β) (Wang et al. (2019); He et al. (2022)). In addition, Zhang et al. (2014b) and Wang et al. (2020a) suggested that Icariin could inhibit the expression of the microglial marker Iba-1 in AD mice. The presence of aberrant protein *A*β accumulation might exacerbate neuroinflammation (Zenaro et al., 2017). The amount of *A*β and tau protein aggregation and the degree of proinflammatory microglial M2 polarization were positively correlated in AD patients (Nordengen et al., 2019). It is believed that neuroinflammation might enhance the formation of NFTs in AD patients (Kinney et al., 2018). Zhang et al. (2014b) revealed that Icariin markedly reduced TGF-1 immunoreactivity, microglial activation, and Aβ deposition at amyloid plaques.

### 3.4 Methodological quality assessment

**FIGURE 3:**
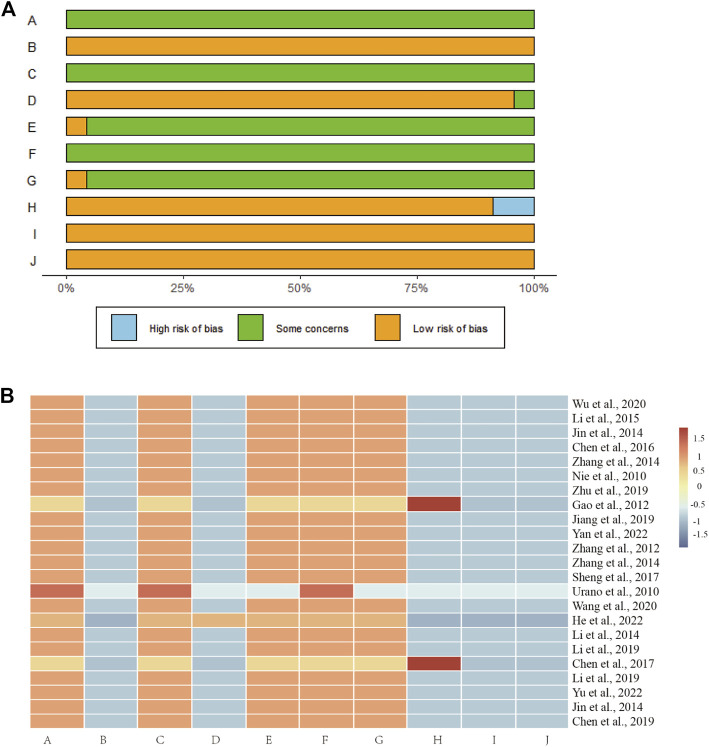
Risk of bias for the 23 included studies. **(A)** Risk of bias histograms for the 23 included studies. **(A)**, sequence generation; **(B)**, baseline characteristics; **(C)**, allocation concealment; **(D)**, animal placement randomization; **(E)**, blinding for animal breeders and researchers; **(F)**, randomized outcome assessment; **(G)**, blinding for outcome raters; **(H)**, incomplete outcome data; **(I)**, optional outcome reporting; and **(J)**, other sources of bias. **(B)** Risk of bias heatmap for each included study. The included studies had poor quality sequence generation, allocation concealment, blinding for animal breeders and researchers, randomized outcome assessment, and blinding for outcome raters.

The work that is performed in animal laboratories serves as a vital link between fundamental science and clinical trials. However, there are a number of confounding variables in animal studies that distort data and undermine their scientific validity. Currently, SYRCLE’s Risk of Bias Tool for Animal Studies is a specific tool for evaluating the authenticity of animal studies (Ferreira et al., 2021), and it includes six items: sequence generation, baseline characteristics, allocation concealment, animal placement randomization, blinding for animal breeders and researchers, randomized outcome assessment, blinding for outcome raters, incomplete outcome data, optional outcome reporting, and other sources of bias. As shown in Figure 3, the included studies had poor quality sequence generation, allocation concealment, blinding for animal breeders and researchers, randomized outcome assessment, and blinding for outcome raters.

### 3.5 The pharmacological properties of icariin

The pharmacological properties of Icariin and its 2D and 3D structures are shown in Table 3. Analysis of the Protox II database showed that Icariin exhibited no hepatotoxicity, carcinogenicity, mutagenicity, or cytotoxicity. The predicted LD50 of Icariin is 5,000 mg/kg, which is quite high.

**TABLE 3:** The pharmacological properties and toxicity report of Icariin

Formula	C33H40O15	TPSA	238.20 Å^2^
MW	676.66 g/mol	Bioavailability Score	0.17
Hato	48	Lipophilicity	0.69
a.Hato	16	BBB	−3
Rbon	9	MR	167.28
Hacc	15	Predicted LD50	5,000 mg/kg
Hdon	8		
Hepatotoxicity	Inactive	Mutagenicity	Inactive
Carcinogenicity	Inactive	Cytotoxicity	Inactive
Immunotoxicity	Active		
2D structure	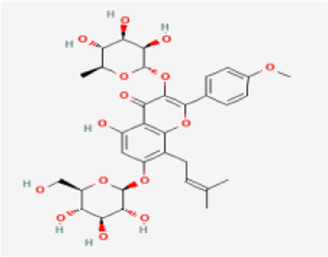	3D structure	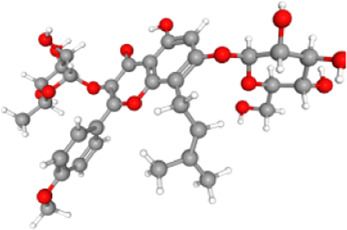

MW, molecular weight; Hato, Num. Heavy atoms; a.Hato, Num. Arom. Heavy atoms; Rbon, number of rotatable bonds; Hacc, number of hydrogen bond acceptors; Hdon, number of hydrogen bond donors; MR, molar refractivity; TPSA, toplogical polar surface area; BBB, blood–brain barrier.

### 3.6 Identification of the therapeutic targets of icariin in AD

**FIGURE 4:**
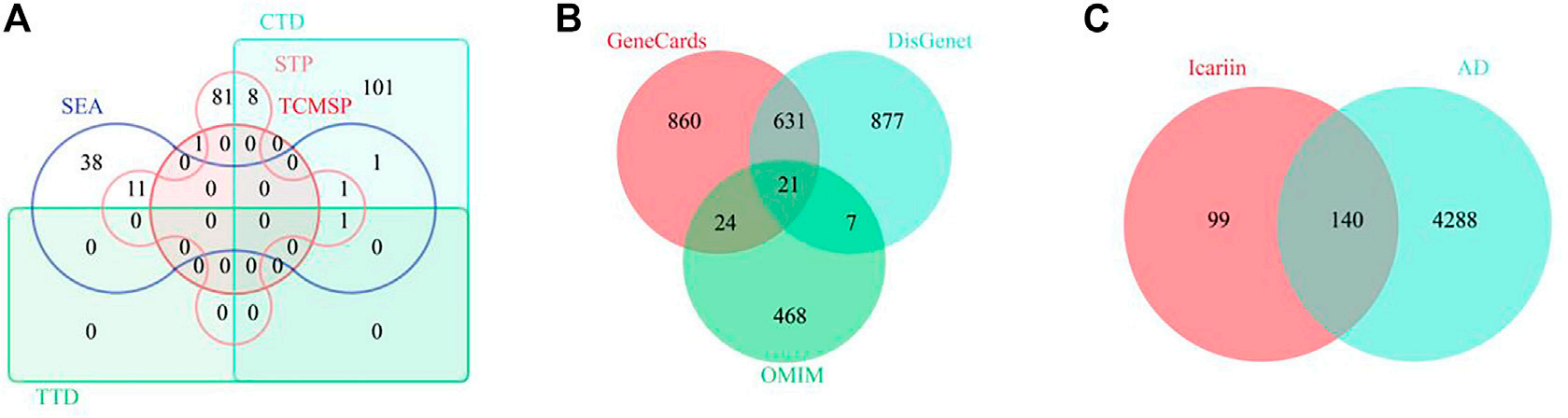
The screening of common targets between Icariin and AD. **(A)** The Venny diagram of Icariin’s targets. A total of 239 targets were collected. **(B)** The Venny diagram of AD’s targets. A total of 4,428 targets were collected. **(C)** The Venny diagram identified 140 intersection targets of Icariin and AD.

To identify the potential targets of Icariin, 1 target from the TCMSP database, 112 targets from the CTD database, 1 target from the TTD database, 54 targets from the SEA database, and 10 targets from the STP database were extracted. After deleting 31 duplicate targets, 239 targets of Icariin were identified (Figure 4A). To identify potential targets in AD, 1,536 targets from the GeneCards database, 3,397 targets from the DisGenet database, and 520 targets from the OMIM database were extracted. After deleting 6,868 duplicate targets, 4,429 potential targets of AD were determined (Figure 4B). Venn diagram analysis identified 140 intersecting targets (Figure 4C).

### 3.7 Analysis of DO enrichment and the protein‒protein interaction network of targets

**FIGURE 5:**
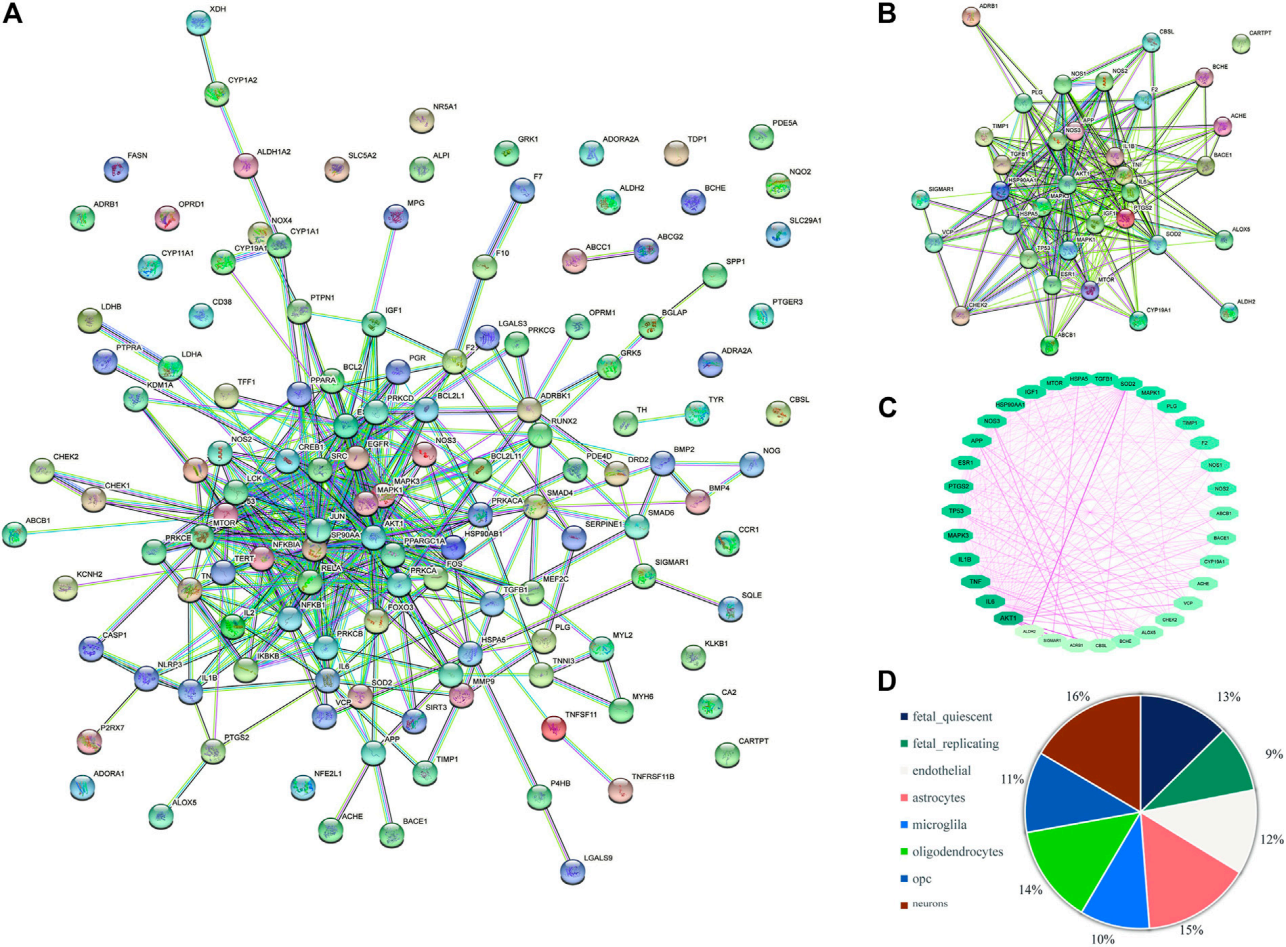
The PPI network of 30 AD related targets. **(A)** The PPI network for 140 intersection targets. **(B)** The PPI network for 35 AD related hub targets. **(C)** The degree change for 34 AD related hub targets. The darker the colour and the larger the font are, the greater the degree value of the target is. **(D)** The distribution of 35 AD related targets in CNS cells.

After performing DO enrichment analysis with the 140 intersection targets *via* the R language, 630 diseases were identified (Supplementary Material S3), and 35 identical targets were identified and shared by AD and tauopathy (Table 4). The 140 overlapping targets between Icariin and AD with the greatest confidence were integrated into the STRING database to generate the PPI network (Figure 5A). The 35 AD-related hub targets were then utilized to create a PPI network (Figure 5B), and the results indicated that CARTPT has no direct connection with other targets. The PPI network with 34 AD hub targets was then imported into Cytoscape for topological parameter analysis. The darker the colour and the larger the font are, the greater the degree value of the target is (Figure 5C). The topological parameters for each target were provided in (Supplementary Material S4). To investigate the cellular distribution of the 35 targets in the human brain, the Single Cell Expression function in AlzData was used. According to Figure 5D, 74.29% of the targets were found in foetal quiescent cells, 54.29% in foetal replicating cells, 68.57% in endothelial cells, 88.57% in astrocytes, 57.14% in microglia, 80.00% in oligodendrocytes, 65.71% in oligodendrocyte precursor cells (opc), and 97.14% in neurons. We further explored the ageing-related targets among the 35 hub targets since AD is an age-related neurodegenerative disorder. According to the data retried from the Aging Atlas, AKT1, TNF, SOD2, APP, IL1B, IL6, MAPK3, MTOR, PTGS2, IGF1, TGFB1, TIMP1, TP53, VCP, HSP90AA1, ESR1, CHEK2, and ALDH2 were age-related.

**TABLE 4:** The DO information for AD and tauopathy

Description	Gene ratio	*p*-value	p.Adjust	q value	Gene symbol	Count
AD	0.26	2.31749E-15	8.07689E-14	2.57499E-14	AKT1/IL6/TNF/MAPK3/TP53/PTGS2/IL1B/ESR1/APP/NOS3/HSP90AA1/IGF1/MTOR/TGFB1/HSPA5/SOD2/MAPK1/PLG/TIMP1/F2/NOS2/NOS1/ABCB1/BACE1/CYP19A1/ACHE/VCP/CHEK2/ALOX5/BCHE/CBSL/ADRB1/SIGMAR1/ALDH2	35
tauopathy	0.26	3.08385E-15	9.67301E-14	3.08385E-14	AKT1/IL6/TNF/MAPK3/TP53/PTGS2/IL1B/ESR1/APP/NOS3/HSP90AA1/IGF1/MTOR/TGFB1/HSPA5/SOD2/MAPK1/PLG/TIMP1/F2/NOS2/NOS1/ABCB1/BACE1/CYP19A1/ACHE/VCP/CHEK2/ALOX5/BCHE/CBSL/ADRB1/SIGMAR1/ALDH2	35

### 3.8 Analysis of GO enrichment

**FIGURE 6:**
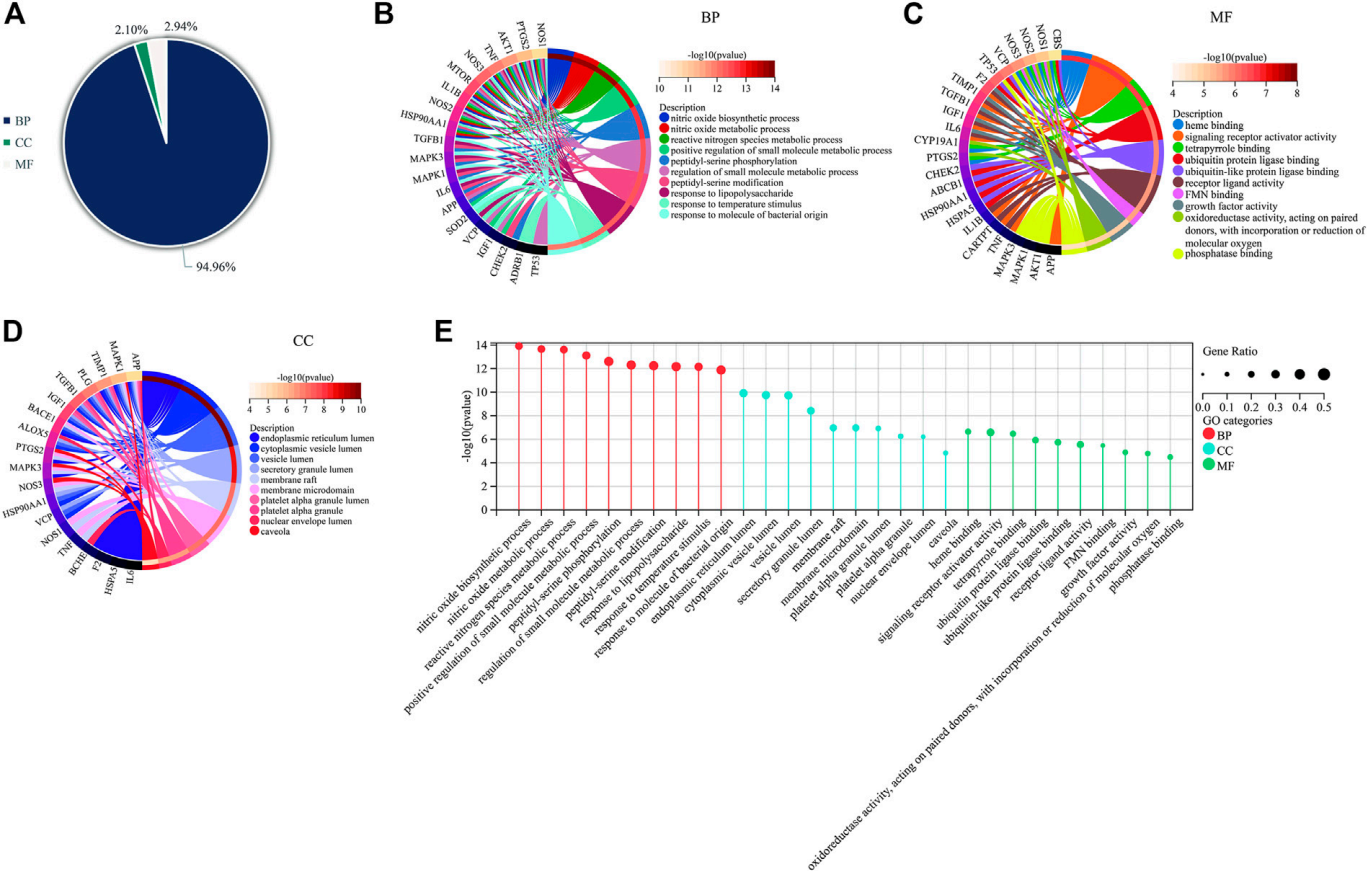
GO enrichment for Icariin’s treatment against VD. **(A)** The proportional distribution diagram of GO items with 94.96% BP, 2.10% CC, and 2.94% MF, respectively. **(B)** The Circro diagram of BP with the top 10 items. **(C)** The Circro diagram of MF with the top 10 items. **(D)** The Circro diagram of CC with the top 10 items. **(E)** The bar diagram was used to display all of the top 10 items for BP, MF, and CC.

The 35 hub targets of Icariin in the treatment of AD were subjected to GO enrichment analysis. A total of 1,668 items were identified, with 1584 BP items (94.96%), 35 CC items (2.10%), and 49 MF items (2.94%) (Figure 6A). The top ten BP terms identified by GO enrichment analysis (Figure 6B) were nitric oxide biosynthetic process, nitric oxide metabolic process, reactive nitrogen species metabolic process, positive regulation of small molecule metabolic process, peptidyl-serine phosphorylation, regulation of small molecule metabolic process, peptidyl-serine modification, response to lipopolysaccharide, response to temperature stimulus, and response to molecule of bacterial origin. The top 10 MF terms identified by GO enrichment analysis (Figure 6C) were haem binding, signalling receptor activator activity, tetrapyrrole binding, ubiquitin protein ligase binding, ubiquitin-like protein ligase binding, receptor ligand activity, FMN binding, growth factor activity, oxidoreductase activity, acting on paired donors, incorporation or reduction of molecular oxygen, and phosphatase binding. The top ten CC terms identified by GO enrichment analysis (Figure 6D) were endoplasmic reticulum lumen, cytoplasmic vesicle lumen, vesicle lumen, secretory granule lumen, membrane raft, membrane microdomain, platelet alpha granule lumen, platelet alpha granule, nuclear envelope lumen, and caveola. The top 10 items for BP, MF, and CC are illustrated *via* bar diagrams (Figure 6E).

### 3.9 Analysis of KEGG pathway enrichment

**FIGURE 7:**
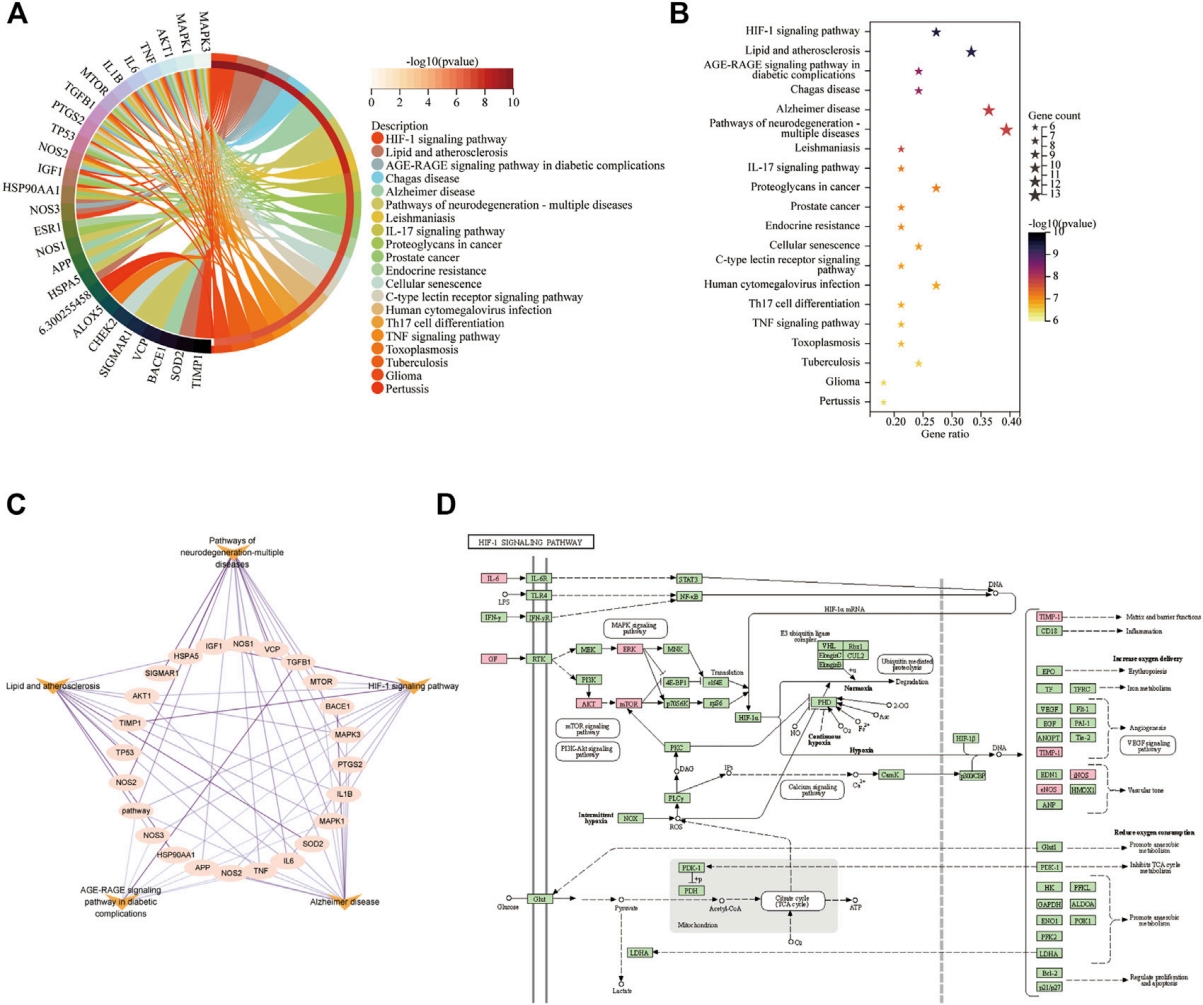
KEGG pathway enrichment for Icariin’s treatment against AD. **(A)** Circro diagram was used to illustrate the top 20 pathways. **(B)** Bubble diagram was used to illustrate the top 20 pathways. **(C)** The network of twenty-two targets involved in HIF-1 signalling pathway, lipid and atherosclerosis, AGE-RAGE signalling pathway in diabetic complications, AD, and pathways of neurodegeneration-multiple diseases pathways. **(D)** HIF-1 signalling pathway. The pink colour targets were the hub targets involved in Icariin’s therapeutic effect on AD.

The 35 hub targets were used to analyse KEGG pathway enrichment. A total of 146 items of the KEGG pathway were identified. The top 20 KEGG signalling pathways (Figures 7A,B) were the HIF-1 signalling pathway, lipid and atherosclerosis, AGE-RAGE signalling pathway in diabetic complications, Chagas disease, AD, pathways of neurodegeneration-multiple diseases, leishmaniasis, IL-17 signalling pathway, proteoglycans in cancer, prostate cancer, endocrine resistance, cellular senescence, C-type lectin receptor signalling pathway, human cytomegalovirus infection, Th17-cell differentiation, TNF signalling pathway, toxoplasmosis, tuberculosis, glioma, and pertussis. Among the top 6 pathways (Table 5), the HIF-1 signalling pathway, lipid and atherosclerosis, AGE-RAGE signalling pathway in diabetic complications, AD, and pathways of neurodegeneration-multiple diseases were used to construct the network. Twenty-two targets were distributed in these five pathways: AKT1, NOS3, NOS2, IL6, MAPK1, MAPK3, MTOR, IGF1, TIMP1, TNF, SOD2, IL1B, HSPA5, TP53, HSP90AA1, TGFB1, APP, PTGS2, BACE1, NOS1, VCP, and SIGMAR1 (Figure 7C). The KEGG mapper was used to describe the hub targets in the HIF-1 signalling pathway (Figure 7D).

**TABLE 5:** The top 6 KEGG pathways

Pathway	Gene ratio	-log10 (*p*-value)	Targets
HIF-1 signalling pathway	0.27	9.52	AKT1; NOS3; NOS2; IL6; MAPK1; MAPK3; MTOR; IGF1; TIMP1
Lipid and atherosclerosis	0.33	9.39	AKT1; NOS3; TNF; SOD2; IL1B; IL6; MAPK1; MAPK3; HSPA5; TP53; HSP90AA1
AGE-RAGE signalling pathway in diabetic complications	0.24	8.36	AKT1; NOS3; TNF; IL1B; IL6; MAPK1; MAPK3; TGFB1
Chagas disease	0.24	8.3	AKT1; NOS2; TNF; IL1B; IL6; MAPK1; MAPK3; TGFB1
Alzheimer disease	0.36	7.82	AKT1; NOS2; TNF; APP; IL1B; IL6; MAPK1; MAPK3; MTOR; PTGS2; BACE1; NOS1
Pathways of neurodegeneration-multiple diseases	0.39	7.79	NOS2; TNF; APP; IL1B; IL6; MAPK1; MAPK3; MTOR; PTGS2; HSPA5; NOS1; VCP; SIGMAR1

### 3.10 Analysis of molecular docking

**FIGURE 8:**
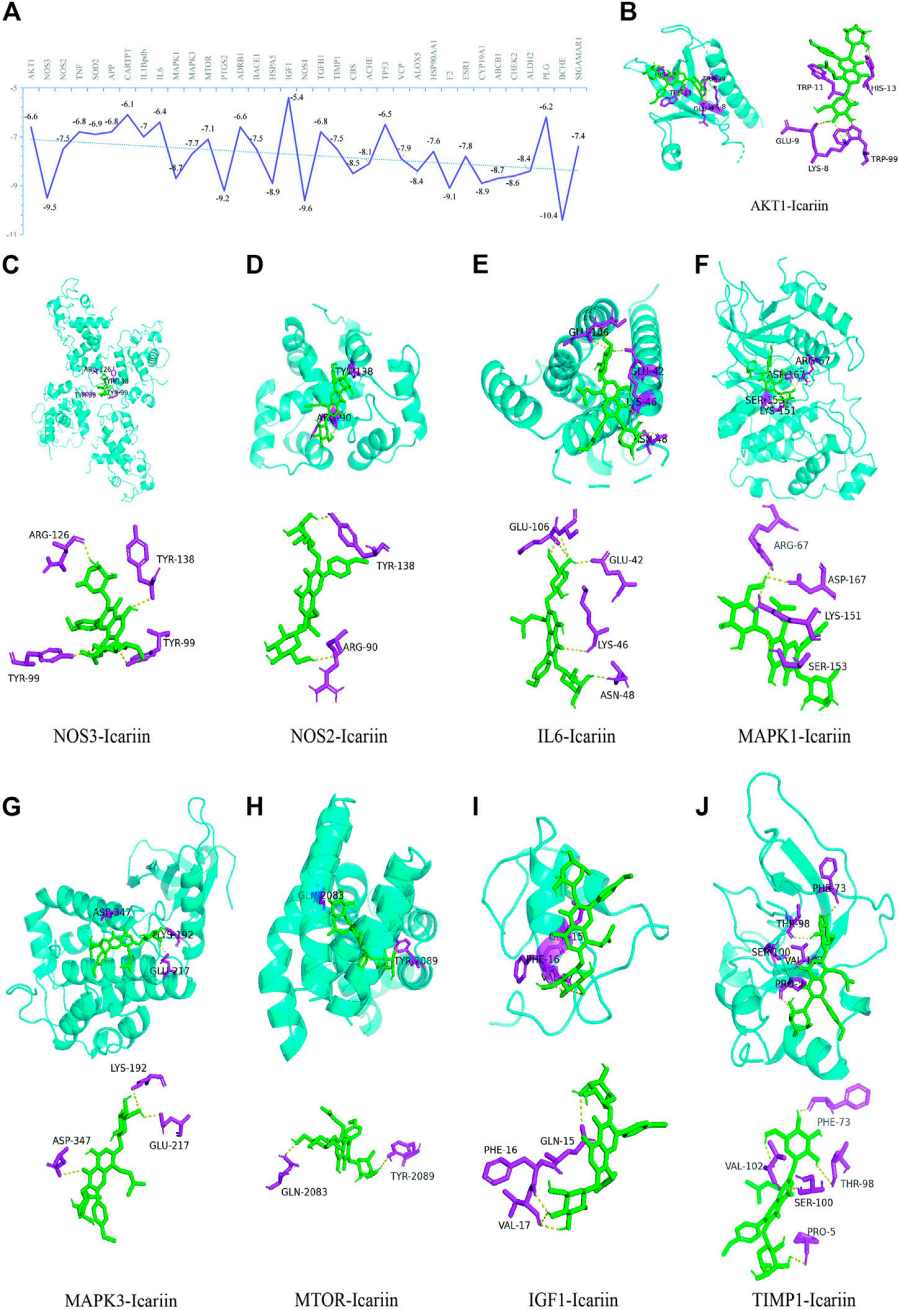
The illustrations of molecular docking. **(A)** The energy docking results of molecular docking. Icariin with AKT1 **(B)**, NOS3 **(C)**, NOS2 **(D)**, IL6 **(E)**, MAPK1 **(F)**, MAPK3 **(G)**, MTOR **(H)**, IGF1 **(I)**, and TIMP1 **(J)**.

As indicated in Figure 8A, the binding energies between the 35 targets and Icariin were between −10.4 and −5.4 kcal/mol. As a binding energy less than −5 kcal/mol indicates stable binding (Gaillard, 2018), 35 hub targets exhibited good binding with Icariin. In the current study, the HIF-1 signaling pathway was the top-ranked pathway, and here, we show the molecular docking results of the nine targets in the HIF-1 signalling pathway (Figures 8B–J).

### 3.11 Molecular docking and molecular dynamics simulation analysis of icariin with HIF-1α

Molecular docking results indicated that the energy of docking between Icariin and HIF-1α was −10.1 kcal/mol. The parameters of the docking box are X-centre: −7.83, Y-centre: 0.871, and Z-centre: −1.479.

**FIGURE 9:**
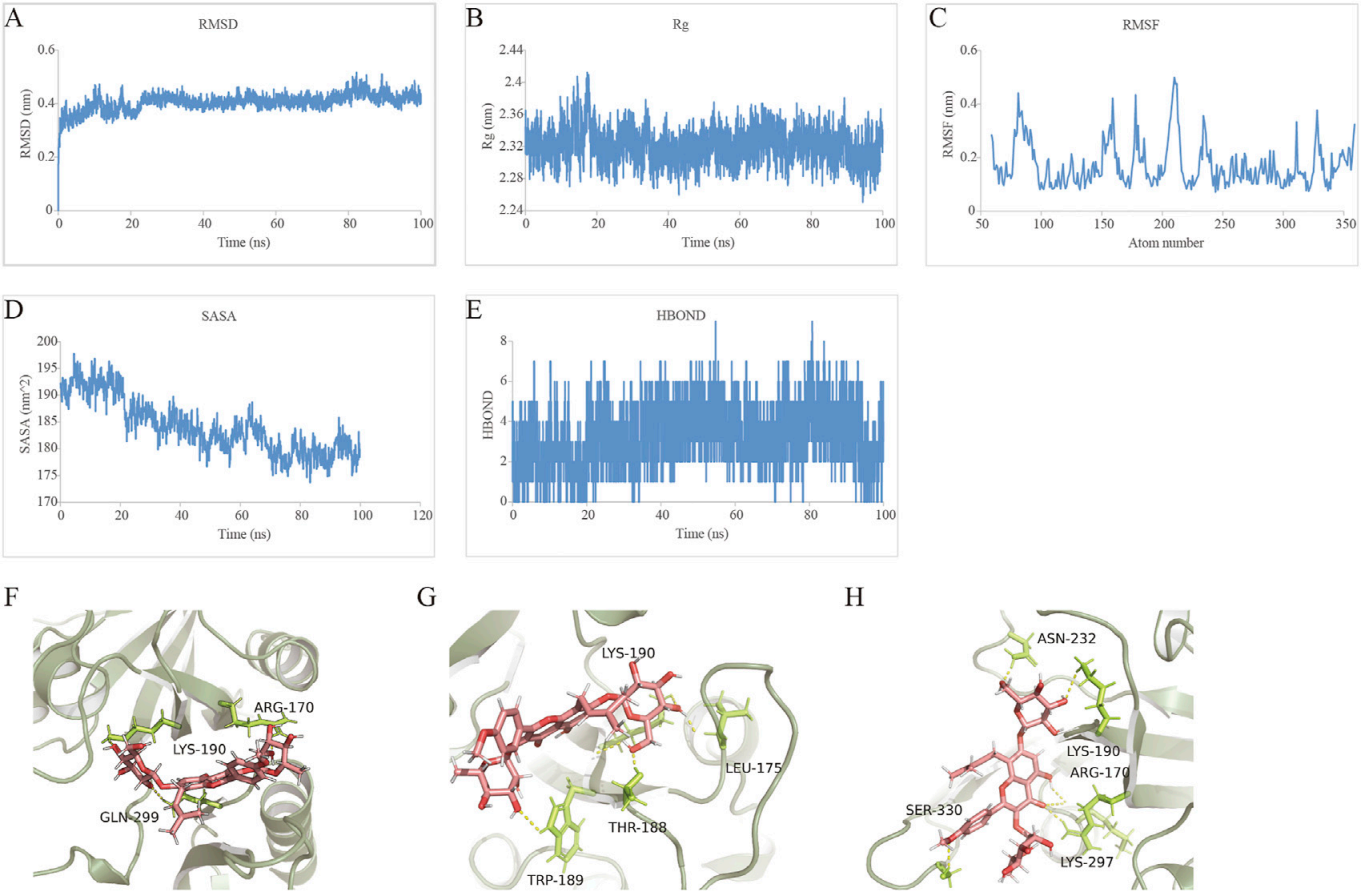
Profiles of MDS. **(A)** The RMSD analysis for the Icariin-HIF-1α complex. **(B)** The Rg analysis for the tightness of Icariin-HIF-1α complex. **(C)** The RMSF analysis for the Icariin-HIF-1α complex. **(D)** The SASA analysis for the compactness of Icariin-HIF-1α complex in the process of induced fit. **(E)** The HBOND analysis for the relationship between the number of hydrogen bonds and the simulation time. **(F)** The hydrogen bond fluctuation at 0 ns **(G)** The hydrogen bond fluctuation at20 ns **(H)** The hydrogen bond fluctuation at 100 ns.

MDS is an important method for studying the stability and kinetic characteristics of complexes in aqueous solution. The root-mean-square deviation (RMSD) was used to measure the stability of the Icariin-HIF-1α system. The RMSD value increased slowly at 0–15 ns, fluctuated slightly at 15–25 ns, remained stable at 25–75 ns, increased slightly at 75 ns, and then quickly decreased and stabilized at 0.4 nm. Root mean square fluctuation (RMSF) can be used to analyse the local site allostery of the complex. The fluctuation of the Icariin-HIF-1α system corresponded to the fluctuation in the RMSD, and a peak of 0–15 ns in the RMSF (Figure 9C) corresponded to the RMSD (Figure 9A), which then stabilized to 75–100 ns and then decreased slowly.

The radius of gyration (Rg) is an important indicator for evaluating the compactness of the system structure, and it can be used to determine the local site allosteric situation of a system during the simulation process. Taking 0.3 nm as the cut-off value, the amino acids at positions 78, 81–91, 157–160, 178, 180, 206–214, 234–235, 311, 328, and 359 underwent local site allostery (Figure 9B).

SASA analysis revealed a steady decline in SASA from 0 to 100 ns, indicating favourable binding and progressive protein tightening (Figure 9D).

HBOND can be used to describe the relationship between the number of hydrogen bonds in the Icariin-HIF-1α complex and the simulation time, and the side shows that ligand recognition played a role in specific recognition. There are 2-4 hydrogen bonds in the stable-state complex (Figures 9D,E). The binding free energy decomposition of the Icariin-HIF-1α complex is shown in Table 6.

**TABLE 6:** Binding free energy decomposition of the Icariin-HIF-1α complex

VDWAALS	EEL	EGB	ESURF	GGAS	GSOLV	TOTAL
−46.159	−39.3252	56.09297	−6.63406	−85.4839	49.4595	−36.0244

VDWAALS, van der Waals energy; Eel, Electrostatic energy; EGB, polar solvation energy; ESURF, Non-polar solvation energy; GGAS, total gas phase free energy; GSOLV, total solvation free energy; TOTAL, GSOLV + GGAS.

Finally, we investigated the stable posture. At 0 ns, the head, middle and tail of Icariin all made contact with HIF-1α, and the corresponding amino acids were ARG-170, LYS-190, and GLN-299, respectively (Figure 9F). At 20 ns, both RMSD and Rg fluctuated significantly. At this time, the HIF-1α cavity was slightly enlarged, and Icariin was expanded. The head of Icariin contacted ASN-232 and LYS-190, and the middle part was in contact with ARG-170 and LYS-297 touch, tail and SER-330 contact (Figure 9G). As the system gradually stabilized, Icariin stabilized in the upper part of the HIF-1α pocket at 100 ns, the head maintained contact with LYS-190, and LEU-175 was located on the surface of the pocket for further contact. THR-188 and TRP-189 inside the pocket fixed the small molecule tail (Figure 9H).

## 4 Discussion

This study’s literature review, which was based on a preclinical trial, compiled the most recent information on the subject and outlined the neuroprotective effects of Icariin on AD. After a thorough and in-depth analysis of the potential targets of Icariin in treating AD, the HIF-1 pathway was shown to be the top ranked signaling pathway. Icariin and HIF-1α may combine to maintain HIF-1α in a stable state, according to the MDS results.

AD is a neurodegenerative disorder that affects over 35 million individuals worldwide. There is a serious and desperate need for the discovery of efficient treatments since AD prevalence is rapidly increasing. Five medications, including acetylcholinesterase inhibitors and N-methyl-d-aspartate receptor antagonists that have been approved by the Food and Drug Administration, primarily target symptom relief and have the potential to cause adverse effects (Joe and Ringman, 2019; Moss and Perez, 2021). Currently, the use of natural compounds as an alternate treatment for AD has been advocated. Clinical studies have demonstrated that traditional Chinese medicine (TCM) could be effective in the amelioration of cognitive impairment in AD patients (Lai et al., 2017; Yang et al., 2017; Pei et al., 2020). Icariin, which is the active chemical component of *Epimedium*, is delivered by blood and can cross the BBB to exert an effect on the CNS (Khezri and Ghasemnejad-Berenji, 2022). The beneficial effects of Icariin on AD have received a great deal of attention in recent years. As a systematic review with meta-analysis indicated in this study, Icariin treatment could improve memory and learning function by decreasing the expression of Aβ_1-40_, Aβ_1-42_, BACE1, tau, and hyperphosphorylated tau, which are critical mediators for the progression of AD. Aβ is the major activator of microglia, which respond to Aβ instantly by migrating towards plaques and phagocytosing Aβ (Bolmont et al., 2008; Baik et al., 2016). Wang et al. (2019) found that Icariin could ameliorate cognitive impairment in AD by controlling the balance between proinflammatory and anti-inflammatory mediators by regulating the NF-κB signalling pathway. Autophagy was also reported to be involved in the neuroprotective effect of Icariin against AD. Combination treatment with β-asarone and Icariin inhibited cell and mitochondrial damage by inducing autophagy/mitophagy in AD models (Wang et al., 2021a). Icariin enhances autophagy‒lysosome function to alleviate amyloid pathologies and tau phosphorylation in AD models (Chen et al., 2016b; Jiang et al., 2019), and these effects could be regulated by the AMPK/mTOR/ULK1 (Zheng et al., 2021) and PI3K/AKT signalling pathways (Sheng et al., 2017).

In the current study, the DOSE package of the R language was used to conduct disease enrichment analysis on the 140 common targets of Icariin and AD, and 35 identical targets were shared by AD and tauopathy. Tauopathy refers to a wide range of phenotypically diverse diseases that are characterized by the aberrant aggregation of tau, which include AD, progressive supranuclear palsy, corticobasal degeneration, Pick’s disease, dementia pugilistica, and frontotemporal dementia with Parkinsonism linked to chromosome 17 (Strang et al., 2019). Among the 35 AD-related targets, the STRING database showed that CARTPT has no interaction with another 34 targets. The endogenous neuropeptide CART, which is widely expressed in the brain (Couceyro et al., 1997), is encoded by CARTPT. Jin et al. (2015) found that exogenous CART administration improved memory impairment in AD mice. In addition, CART could promote Aβ degradation by regulating Aβ metabolism-associated enzymes (Jiang et al., 2021). Hence, in this study, all 35 AD-related targets, including CARTPT, were considered hub targets for further analysis.

Molecular docking indicated that the 35 hub targets and Icariin docked well. The HIF-1 signalling pathway was revealed to be the most prominent pathway, and this pathway regulates matrix and barrier functions, angiogenesis, and vascular tone, as indicated by KEGG mapper. The hub targets of AKT1, NOS3, NOS2, IL6, MAPK1, MAPK3, MTOR, IGF1, and TIMP1 are components of the HIF-1 signalling pathway. It has been well demonstrated that hypoxia is an important pathology associated with AD (Zhang et al., 2019b). HIF-1 is a heterodimer comprising HIF-1α and HIF-1β subunits, which are function as key regulators of the cell response to hypoxia by mediating the expression of various target genes that regulate both adaptive and pathogenic processes (Wu et al., 2019). Under hypoxic conditions, inactivated oxygen-dependent HIF prolyl hydroxylase leads to HIF-1α stabilization, followed by its translocation into the nucleus to bind to HIF-1β (Yu et al., 2021; Fang et al., 2022) in order to promote the expression of target genes that promote increased oxygen supply by enhancing angiogenesis (Mitroshina et al., 2021). Therefore, HIF-1α is the core target in the initiation of the HIF-1 pathway. Wang YY et al. (Wang et al., 2021b) revealed an intriguing finding that HIF-1 exerted both neuroprotective and negative effects. HIF-1α could enhance Aβ generation by promoting β/γ-secretases and inhibiting α-secretases, as well as inactivating microglia, to promote AD pathogenesis. Although the role of HIF-1 signalling in the development of AD-related neurodegeneration is rather controversial (Chai et al., 2014; Guo et al., 2017; Kim et al., 2017; Lu et al., 2018), increasing evidence has identified HIF-1α as a potential therapeutic target for AD (Wang et al., 2021b). Increased HIF-1 activity and/or increased expression of HIF-1 target proteins could alleviate cognitive impairment and AD progression by regulating glycolysis and capillary blood supply (Iyalomhe et al., 2017). Furthermore, HIF-1α has been shown to prevent tau hyperphosphorylation and neurofilament formation (Liu et al., 2008; Merelli et al., 2018). Icariin is involved in the regulation of various diseases by activating the HIF-1 signalling pathway. Chai X et al. (Zhao et al., 2021) indicated that Icariin could improve testicular dysfunction by activating the SIRT1/HIF-1α pathway in rats with diabetes mellitus. Wang P et al. (Wang et al., 2020b) found that Icariin treatment facilitates chondrocyte vitality by promoting HIF-1α expression and anaerobic glycolysis. Moreover, Icariin could regulate the HIF-1α/CXCR4 pathway to promote the migration of bone marrow stromal cells (Zhu et al., 2018). However, no reports have suggested that Icariin can exert a neuroprotective effect by regulating the HIF-1 signalling pathway. This study revealed that Icariin docked well with the hub targets in the HIF-1 pathway. Next, we further explored the possible effect of Icariin on HIF-1α. The molecular docking between HIF-1α and Icariin indicated that the Icariin-HIF-1α complex was well docked, and the molecular docking energy value of Icariin and HIF-1α was −10.1 kcal/mol. The lower the molecular docking energy, the more stable the complex formed was. Obviously, the molecular docking energy value of Icariin and HIF-1α was the lowest. As the stabilization of HIF-1α might be a promising therapeutic target for AD, HIF-1α could be a potential new target for Icariin’s treatment against AD. By using the MDS method to evaluate the dynamic binding of the complex within 100 ns, the RMSD results suggested that the complex reached a state of equilibrium. The Rg result indicated that the structure of HIF-1α-Icariin was stable. The SASA results indicated that the complex SASA gradually shrank, indicating that the system gradually became stable during the simulation. Therefore, the combination of Icariin and HIF-1α could maintain HIF-1α stability to a certain extent. As stabilization of HIF-1α might be a promising therapeutic target for AD, HIF-1α could be a potential new target for Icariin’s treatment against AD. Hence, we speculated that HIF-1α would be an initial target for Icariin’s neuroprotective role against AD.

Although this study discussed and analysed the role of Icariin in the treatment of AD, some limitations exist. First, negative findings may not have been reported, which could result in information biases. In addition, the included studies were not of good quality and were mainly affected by biases in sequence generation, covert grouping, blinding for animal breeders and researchers, randomized outcome evaluation, and blinding for outcome raters; these biases may affect the accuracy and reliability of the meta-analysis results. This study revealed that the HIF-1 pathway could be a potential pathway by which Icariin plays its neuroprotective role against AD according to computer-aided drug analysis, but this has not been verified experimentally. We plan to measure the expression of HIF-1α in the brains of mice with AD and assess cognitive impairment, Aβ clearance, BBB injury, and angiogenesis in the brain after blocking HIF-1α activity to comprehensively evaluate the possible role of the HIF-1 signalling pathway in the neuroprotective effect of Icariin against AD.

## 5 Conclusion

This study is the first to use the method of systematic review combined with meta-analysis to evaluate and discuss the development of Icariin for use in the treatment of AD. Based on the KEGG pathway enrichment analysis that revealed that the HIF-1 signalling pathway was the top signalling pathway, molecular docking and MDS methods were further used to indicate that HIF-1α could be a potential target of Icariin in the treatment of AD. Although the role of the HIF-1 signalling pathway in the neuroprotective effect of Icariin on AD has not been verified experimentally, this work identified a new prospective target of Icariin in the treatment of AD.

## Data Availability

The datasets presented in this study can be found in online repositories. The names of the repository/repositories and accession number(s) can be found in the article/Supplementary Material.
