# Supplementary material for: HIF-1 signalling pathway was identified as a potential new pathway for Icariin’s treatment against Alzheimer’s disease based on preclinical evidence and bioinformatics
Source: Front Pharmacol. 2022 Dec 1;13:1066819. doi: 10.3389/fphar.2022.1066819 (PMC9751333; doi:10.3389/fphar.2022.1066819)
Supplement: Supplementary file 3 [file Table1.DOCX]

**Supplementary material 1: The search strategy for article retrieval about Icariin's treatment against AD**

1. (3-((6-deoxymannopyranosyl)oxy)-7-(glucopyranosyloxy)-5-hydroxy-2-(4-methoxyphenyl)-8-(3-methyl-2-butenyl)-4H-1-benzopyran-4-one) OR (icariin)
2. ((((((((((((((((((((((((((((((((((Alzheimer Dementia) OR (Alzheimer Dementias)) OR (Dementia, Alzheimer)) OR (Alzheimer's Disease)) OR (Dementia, Senile)) OR (Senile Dementia)) OR (Dementia, Alzheimer Type)) OR (Alzheimer Type Dementia)) OR (Alzheimer-Type Dementia (ATD))) OR (Alzheimer Type Dementia (ATD))) OR (Dementia, Alzheimer-Type (ATD))) OR (Alzheimer Type Senile Dementia)) OR (Primary Senile Degenerative Dementia)) OR (Dementia, Primary Senile Degenerative)) OR (Alzheimer Sclerosis)) OR (Sclerosis, Alzheimer)) OR (Alzheimer Syndrome)) OR (Alzheimer's Diseases)) OR (Alzheimer Diseases)) OR (Alzheimers Diseases)) OR (Senile Dementia, Alzheimer Type)) OR (Acute Confusional Senile Dementia)) OR (Senile Dementia, Acute Confusional)) OR (Dementia, Presenile)) OR (Presenile Dementia)) OR (Alzheimer Disease, Late Onset)) OR (Late Onset Alzheimer Disease)) OR (Alzheimer's Disease, Focal Onset)) OR (Focal Onset Alzheimer's Disease)) OR (Familial Alzheimer Disease (FAD))) OR (Alzheimer Disease, Familial (FAD))) OR (Familial Alzheimer Diseases (FAD))) OR (Alzheimer Disease, Early Onset)) OR (Early Onset Alzheimer Disease)) OR (Presenile Alzheimer Dementia)
3. ((3-((6-deoxymannopyranosyl)oxy)-7-(glucopyranosyloxy)-5-hydroxy-2-(4-methoxyphenyl)-8-(3-methyl-2-butenyl)-4H-1-benzopyran-4-one) OR (icariin)) AND (((((((((((((((((((((((((((((((((((Alzheimer Dementia) OR (Alzheimer Dementias)) OR (Dementia, Alzheimer)) OR (Alzheimer's Disease)) OR (Dementia, Senile)) OR (Senile Dementia)) OR (Dementia, Alzheimer Type)) OR (Alzheimer Type Dementia)) OR (Alzheimer-Type Dementia (ATD))) OR (Alzheimer Type Dementia (ATD))) OR (Dementia, Alzheimer-Type (ATD))) OR (Alzheimer Type Senile Dementia)) OR (Primary Senile Degenerative Dementia)) OR (Dementia, Primary Senile Degenerative)) OR (Alzheimer Sclerosis)) OR (Sclerosis, Alzheimer)) OR (Alzheimer Syndrome)) OR (Alzheimer's Diseases)) OR (Alzheimer Diseases)) OR (Alzheimers Diseases)) OR (Senile Dementia, Alzheimer Type)) OR (Acute Confusional Senile Dementia)) OR (Senile Dementia, Acute Confusional)) OR (Dementia, Presenile)) OR (Presenile Dementia)) OR (Alzheimer Disease, Late Onset)) OR (Late Onset Alzheimer Disease)) OR (Alzheimer's Disease, Focal Onset)) OR (Focal Onset Alzheimer's Disease)) OR (Familial Alzheimer Disease (FAD))) OR (Alzheimer Disease, Familial (FAD))) OR (Familial Alzheimer Diseases (FAD))) OR (Alzheimer Disease, Early Onset)) OR (Early Onset Alzheimer Disease)) OR (Presenile Alzheimer Dementia)).
