# Supplementary figures and images for: HIF-1 signalling pathway was identified as a potential new pathway for Icariin’s treatment against Alzheimer’s disease based on preclinical evidence and bioinformatics
Source: Front Pharmacol. 2022 Dec 1;13:1066819. doi: 10.3389/fphar.2022.1066819 (PMC9751333; doi:10.3389/fphar.2022.1066819)

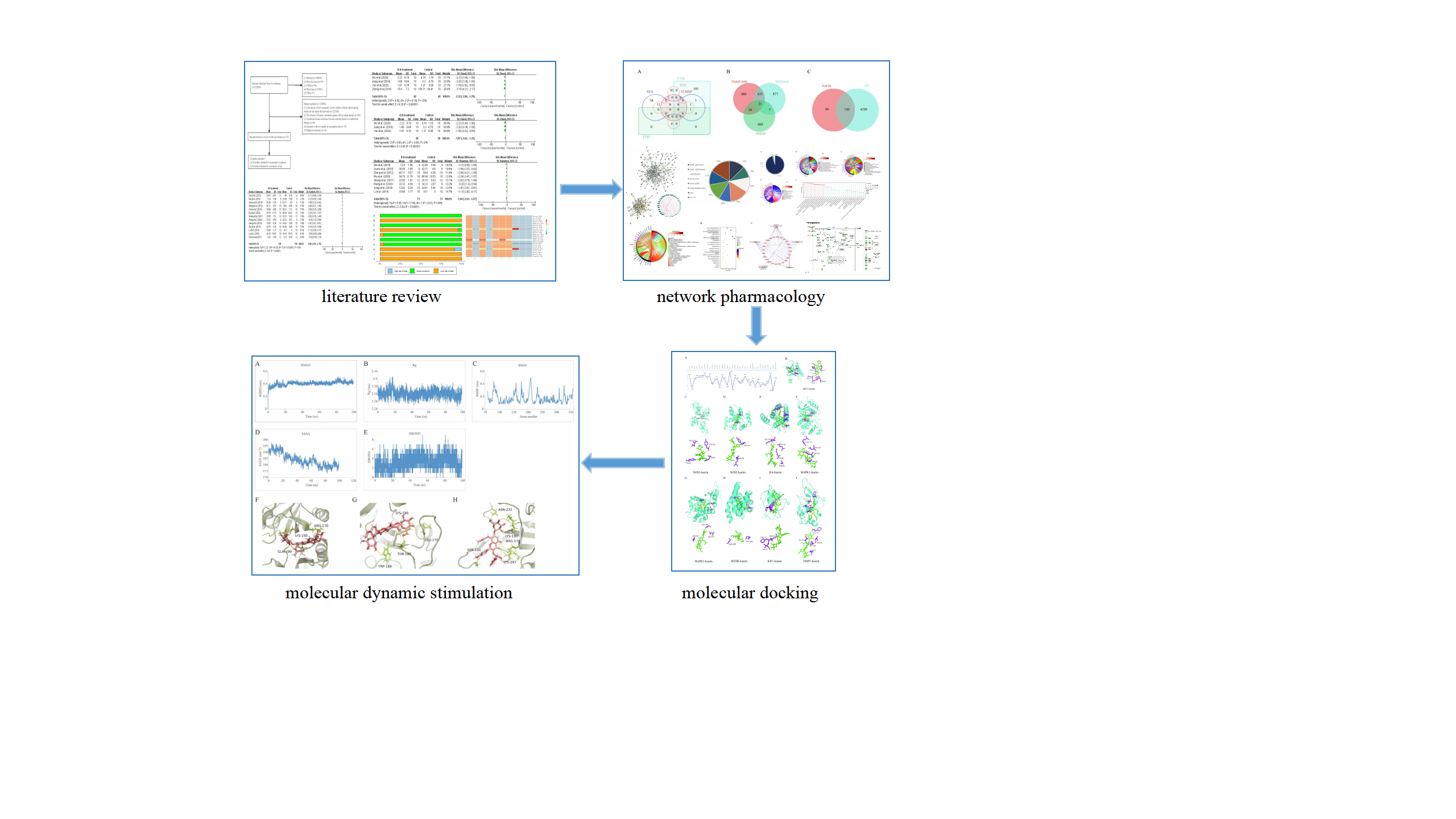

Supplement: Supplementary file 4 [file Image1.TIF]
